# Long-Term Risk of Cardiovascular Disease among Type 2 Diabetic Patients with Asymptomatic Intracranial Atherosclerosis: A Prospective Cohort Study

**DOI:** 10.1371/journal.pone.0106623

**Published:** 2014-09-05

**Authors:** Jian Gang Duan, Xiang Yan Chen, Alex Lau, Adrian Wong, G. Neil Thomas, Brian Tomlinson, Roxanna Liu, Juliana C. N. Chan, Thomas W. Leung, Vincent Mok, Ka Sing Wong

**Affiliations:** Department of Emergency, Xuanwu Hospital, Capital Medical University, Beijing, China; Department of Medicine and Therapeutics, Chinese University of Hong Kong, Prince of Wales Hospital, Shatin, Hong Kong SAR, China; Department of Psychological Studies and Center for Psychosocial Health and Aging, The Hong Kong Institute of Education, Hong Kong SAR, China; Department of Public Health, Epidemiology and Biostatistics, University of Birmingham, Birmingham, United Kingdom; VCU, United States of America

## Abstract

**Objective:**

To investigate whether asymptomatic middle cerebral artery (MCA) stenosis is associated with risk of cardiovascular disease (CVD) in Chinese with type 2 diabetes.

**Methods:**

In this prospective cohort study, 2,144 Hong Kong Chinese with type 2 diabetes and without history of stroke or atrial fibrillation were recruited in 1994–1996 and followed up for a median of 14.51 years. Participants were assessed at baseline for MCA stenosis using transcranial Doppler. We performed survival analysis to assess the association between asymptomatic MCA stenosis and first CVD event, defined as ischemic stroke, acute coronary syndrome (ACS) or cardiovascular death.

**Results:**

Of the 2,144 subjects, MCA stenosis at baseline was detected in 264 (12.3%). Rates of stroke, ACS and cardiovascular death per 100 were, respectively, 2.24, 2.92 and 1.11 among participants with stenosis, higher than among those without stenosis. Ten-year cumulative occurrence of stroke, ACS and cardiovascular death in subjects with MCA stenosis was 20%, 24% and 10%, respectively, higher than the corresponding values for subjects without stenosis(all P<0.001). After adjusting for covariates, MCA stenosis was found to be an independent predictor of stroke [hazard ratio (HR) 1.40, 95%CI 1.05–1.86; P = 0.02], ACS (HR 1.35, 95%CI 1.04–1.75; P = 0.02) and cardiovascular death(HR 1.56, 95%CI 1.04–2.33; P = 0.03).

**Conclusions:**

Asymptomatic MCA stenosis is a risk factor for CVD in Chinese with type 2 diabetes, and detection of asymptomatic MCA stenosis by transcranial Doppler can identify diabetic individuals at high risk of future CVD. This finding is particularly important for diabetic individuals in Asia, where intracranial atherosclerosis is common.

## Introduction

Cardiovascular disease (CVD), including acute coronary syndrome (ACS) and ischemic stroke, is responsible for 30% of all deaths worldwide, most of which occur in developing countries [1]. Type 2 diabetes shows a strong association with various CVD. Individuals with type 2 diabetes are at 2- to 4-fold higher risk of CVD mortality than those without diabetes [2]. In fact, CVD, particularly coronary artery disease (CAD) resulting from accelerated atherosclerosis, is the leading cause of morbidity and mortality among people with diabetes, causing more deaths than microvascular complications. In addition, diabetes and ischemic stroke often occur together [3]. Therefore improving our ability to identify individuals with diabetes likely to develop CVD may improve their prognosis.

The strong association between CVD and type 2 diabetes reflects substantial overlap in their clinical features. Hypertension and dyslipidemia are well-known risk factors for CVD, and both frequently occur in diabetes. Atherosclerosis is the leading cause of CVD, and intracranial atherosclerotic vascular lesions frequently occur in Chinese with diabetes in the form of middle cerebral artery (MCA) stenosis [4]. Symptomatic MCA stenosis, as well as diabetes itself, is significant independent predictors of stroke recurrence and mortality in Asian patients [5]. MCA stenosis can also occur in an asymptomatic form that has traditionally been considered benign [6], [7]. Increasing evidence suggests that asymptomatic MCA stenosis is not benign: it is associated with a more atherogenic metabolic profile [8] and is an independent predictor of vascular mortality [9] in Chinese with type 2 diabetes. These findings suggest that atherosclerotic lesions develop silently over years until they suddenly become symptomatic. This highlights the need to detect MCA stenosis early, while it is still in the asymptomatic phase.

Routine screening for CAD is not recommended for those with asymptomatic diabetes because it is not thought to improve outcomes as long as CVD risk factors are treated [10]. This means that asymptomatic MCA stenosis may go undetected in a potentially substantial proportion of diabetic population, placing them at higher risk of CVD in the future. Here we carried out a prospective cohort study among Hong Kong Chinese with type 2 diabetes to quantify how much asymptomatic MCA stenosis increases risk of ischemic stroke, ACS and cardiovascular death.

## Materials and Methods

The study protocol was approved by the clinical research ethics committee of the Chinese University of Hong Kong. In this prospective cohort study, individuals with type 2 diabetes were recruited from the diabetes clinic at the Prince of Wales Hospital between 1994 and 1996. All participants were Hong Kong Chinese who lived in Hong Kong throughout the study period. Participants gave written, informed consent. None had a history of stroke, transient ischemic stroke (TIA) or atrial fibrillation.

### Baseline Assessment

Patients were considered to have diabetes if the fasting plasma glucose was ≥7.8 mmol/l, if 2-h plasma glucose was ≥11.1 mmol/l after a 75-g oral glucose tolerance test, or if they were using antidiabetic medication at the time of enrollment. Type 1 diabetic patients were defined as those having acute symptoms including heavy ketonuria (over 3+) or ketoacidosis at the original diagnosis, or as those who required continuous insulin treatment within 1 year of diagnosis [11]. These individuals made up a small percentage of those recruited for participation, and they were excluded from the study.

At baseline, participants were examined by transcranial Doppler (TCD, EME TC-2000). Two experienced operators (K.S.W., R.L.) performed all TCD evaluations. We studied the MCA using a standardized protocol and diagnosed MCA stenosis when peak systolic flow velocity was ≥140 cm/s [5]. When diagnosing stenosis, we also took into account the age of the patients, the presence of turbulence or musical sound, and whether the abnormal velocity was segmental. When insonation of the cerebral arteries was not possible through the temporal window, the individual was excluded from analyses. These TCD-based diagnostic criteria have been shown to be as sensitive and specific as diagnosis based on supplementary magnetic resonance angiography (MRA) and clinical outcome [4], [12].

Individuals also provided information at baseline about history of hypertension and ischemic heart disease (IHD), diabetes duration, smoking, and use of medication, including hypoglycemics (e.g. oral hypoglycemics and insulin), anti-hypertensives, aspirin and statin. Either current or former smoking was defined as positive smoking status for the purposes of this study. Peripheral artery disease (PAD) was diagnosed if claudication, gangrene, or ischemia-related amputation was present. After overnight fasting, blood samples were analyzed for fasting blood glucose, hemoglobin A1c (HbA1c), total cholesterol (TC), triglycerides (TG), high-density lipoprotein cholesterol (HDL-C), and low-density lipoprotein cholesterol (LDL-C). Seated blood pressure, body weight, height and waist circumferences were measured in all participants, and body mass index (BMI) was calculated. The albumin-to-creatinine ratio (ACR) in urine was also measured after overnight fasting. Albuminuria was diagnosed if the ACR was ≥3.5 mg/mmol [13]. Retinopathy was assessed by an ophthalmologist at the time of enrollment. Fundi were examined through dilated pupils, and retinopathy was considered to be present if one or more of the following were observed: hemorrhage, microaneurysm, cotton wool spots, and/or laser coagulation scars related to diabetic retinopathy.

### Outcome Assessment

Participants were followed up from enrollment in 1994–1996 until August 2012. The primary end point was the first cardiovascular event, defined as ischemic stroke (including TIA) or ACS. For the purposes of this study, ischemic stroke was defined as being associated with large-artery atherosclerosis based on the TOAST classification scheme. ACS was defined as unstable angina or myocardial infarction with or without ST-segment elevation. Secondary end points were cardiovascular death due to stroke or ACS.

Hospitalization and outcomes of study participants were tracked through the Hong Kong Hospital Authority Central Computer System, which records admissions to, and discharges from, all public hospitals in Hong Kong. Participants were unambiguously identified in this computer system by their unique Hong Kong Identity Card number, which is compulsory for all residents of Hong Kong. First ischemic stroke (including TIA) or ACS were identified based on discharge diagnosis codes 432–436, 410, and 413, according to the International Classification of Diseases, Ninth Revision (ICD-9). Deaths due to ischemic stroke or ACS were identified based on ICD-9 discharge codes of 432–438, 410, or 413. The exact cause of death was determined from death certificates or medical records. When end points for a given participant were uncertain, we also reviewed paper-based medical records or called the patients or their relatives for follow-up.

### Statistical Analysis

Values of normally distributed variables, including age, diabetes duration, and anthropometric and fasting plasma biochemical parameters, are presented as mean ± SD. Length of follow-up is presented as median and interquartile range (IQR). Hemoglobin A1c (HbA1c) levels are reported both as % and as mmol/mol. Values of categorical variables, including sex, smoking, hypertension, PAD, albuminuria, retinopathy, MCA stenosis, IHD history, and use of medications are presented as percentages.

Baseline characteristics stratified by MCA stenosis were compared using χ^2^-squared and independent-samples *t* tests as appropriate. Whether the presence or absence of asymptomatic MCA stenosis predicts stroke, ACS and cardiovascular death was assessed using Kaplan-Meier survival analysis. Cumulative and mean yearly incidences of ischemic stroke, ACS and cardiovascular death were estimated for individuals with type 2 diabetes with and without MCA stenosis using life tables. We further assessed the effect of asymptomatic MCA stenosis on each outcome by calculating event rates per 100 person-years.

Three Cox proportional hazard regression models were constructed to identify whether MCA stenosis can predict long-term clinical outcomes. Results are expressed as hazard ratios (HRs) with associated 95% confidence intervals (CIs). All models to assess association between MCA stenosis and cardiovascular events were adjusted for the following potential confounders: age, sex, smoking, hypertension, total cholesterol, LDL, HDL, PAD, albuminuria, IHD history, diabetes duration, retinopathy, and HbA1c. In the Cox analyses, HbA1c levels were dichotomized as <7% or ≥7% (53 mmol/mol), since this cut-off has been shown to predict risk of macrovascular disease [14].

All statistical analyses were performed using PASW statistics (version 18.0, IBM, Chicago, IL, USA). Two-sided p-values of <0.05 were considered statistically significant.

## Results

During the enrollment period from 1994–1996, 2,585 individuals with diabetes without symptoms of cerebral vascular disease and atrial fibrillation were recruited. Of these, 44 (1.7%) were excluded because they had type 1 disease and 344 (13.3%) were also excluded because insonation of their cerebral arteries through the temporal window proved impossible. Among the remaining 2,197 individuals with type 2 diabetes, 272 (12.4%) were diagnosed with MCA stenosis. Since 53 individuals (2.4%) were lost to follow-up, the final analyses involved 2,144 patients, corresponding to 97.6% of the original 2,197.

MCA stenosis was diagnosed by TCD in 264 (12.3%). Median follow-up was 14.51 years (IQR 12.48–15.62; range 0.26–19.49). Of the 2,144 participants, 353 (16.5%) developed ischemic stroke and 455 (21.2%) developed ACS. A total of 155 (7.2%) died from cardiovascular disease.

### Baseline clinical characteristics

Table 1 summarizes baseline characteristics of all recruited individuals, with and without stratification based on MCA stenosis. Most participants (86.5%) were being treated for hypoglycemia at the time of enrollment. Individuals with stenosis were generally older than those without stenosis, and they had had diabetes longer and their levels of TC and LDL were higher. Prior macro- and microvascular complications were more frequent among those with MCA stenosis. Prior or current use of hypoglycemic or anti-hypertensive medications, aspirin, and statin was more frequent among those with MCA stenosis.

**Table 1:** Baseline characteristics of 2,197 Chinese with type 2 diabetes with and without asymptomatic MCA stenosis.

Characteristic	Overall (n = 2197)	MCA stenosis (n = 272)	No MCA stenosis (n = 1925)	p-value*
**Age, years**	55.4 (11.3)	59.3 (10.8)	54.8 (11.3)	<0.001†
**Male, n(%)**	896 (40.8)	102 (37.5)	794 (41.2)	0.24‡
**BMI, kg/m^2^**	24.9 (3.7)	24.4 (3.6)	24.9 (3.7)	0.03†
**Waist circumference, cm**	84.6 (9.6)	83.6 (9.7)	84.7 (9.6)	0.11†
**Systolic blood pressure, mmHg**	138.4 (21.5)	147.4 (23.1)	137.2 (21.0)	<0.001†
**Dystolic blood pressure, mmHg**	78.9 (11.4)	78.7 (11.9)	78.9 (11.3)	0.82†
**Total cholesterol, mmol/l**	5.6 (1.1)	5.8 (1.3)	5.6 (1.1)	0.02†
**Triglycerides, mmol/l**	1.8 (1.6)	1.7 (1.4)	1.8 (1.7)	0.42†
**LDL cholesterol, mmol/l**	3.6 (0.9)	3.8 (1.1)	3.6 (0.9)	0.003†
**HDL cholesterol, mmol/l**	1.26 (0.36)	1.27 (0.35)	1.26 (0.36)	0.77†
**Fasting glucose, mmol/l**	9.06 (3.5)	9.06 (3.3)	9.06 (3.5)	0.99†
**HbA1c, %**	7.9 (1.9)	7.9 (1.8)	7.8 (1.9)	0.51†
**HbA1c, mmol/mol**	62.3 (20.3)	63.2 (19.8)	62.1 (20.4)	0.44†
**Medical history, n(%)**				
**Smoking**	599 (27.3)	80 (29.4)	519 (27.0)	0.40‡
**Diabetes duration, mo.**	81.1 (72.9)	99.8 (79.5)	78.5 (71.5)	<0.001†
**Hypertension**	1155 (52.6)	195 (71.7)	960 (49.9)	<0.001‡
**IHD history**	62 (2.8)	16 (5.9)	46 (2.4)	0.001‡
**Albuminuria**	659 (30.0)	104 (39.4)	555 (29.7)	0.001‡
**Peripheral arterial disease**	46 (2.1)	14 (5.3)	32 (1.7)	<0.001‡
**Retinopathy**	506 (23.0)	96 (35.3)	410 (21.3)	<0.001‡
**Medication, n(%)**				
**Hypoglycemic therapy**	1901 (86.5)	250 (91.9)	1651 (85.9)	0.007‡
**Aspirin**	32 (1.5)	11 (4.0)	21 (1.1)	<0.001‡
**Antihypertensive therapy**	423 (19.3)	75 (27.6)	348 (18.1)	<0.001‡
**Statin**	112 (5.1)	22 (8.1)	90 (4.7)	0.02‡

Data are mean ± SD or n (%).

BMI, body mass index; ACEI, angiotensin-converting enzyme inhibitors; HbA1c, glycated hemoglobin; HDL, high-density lipoprotein; LDL, low-density lipoprotein; MCA, middle cerebral artery; IHD: ischemic heart disease.

Hypertension was defined as systolic blood pressure ≥140 mmHg or diastolic blood pressure ≥90 mmHg, or the use of ACE inhibitors, angiotensin II receptor blockers, or other antihypertensive drugs.

*P values refer to comparisons between groups with and without MCA stenosis.

† From independent-samples *t* test.

‡ From χ^2^-squared test.

### MCA Stenosis and CVD Incidence

Mean yearly incidences of stroke, ACS and cardiovascular death in individuals with diabetes and MCA stenosis were, respectively, 2.06%, 2.67% and 0.944%, which were higher than the corresponding incidences of 1.42%, 1.67% and 0.47% in those without stenosis. Individuals with diabetes and MCA stenosis lost fewer person-years due to stroke, ACS or cardiovascular death. Rates of stroke, ACS, and cardiovascular death per 100 person-years were higher among participants with MCA stenosis (2.24, 2.92, 1.11) than among those without it (1.22, 1.66, 0.48) (Table 2). As described below, significant associations were observed between the presence of MCA stenosis and the occurrence of stroke, ACS and cardiovascular death during follow-up.

**Table 2:** Rates of stroke, ACS and cardiovascular death among Chinese with type 2 diabetes with or without asymptomatic MCA stenosis.

	No. of events	Median (IQR), yr*	Person-years	Event rate†
***Stroke***				
**MCA stenosis**	64	12.87 (6.1–14.9)	2857	2.24
**No MCA stenosis**	289	14.33 (10.6–15.5)	23765	1.22
***ACS***				
**MCA stenosis**	82	12.60 (5.9–14.9)	2810	2.92
**No MCA stenosis**	373	14.26 (9.8–15.5)	23377	1.6
***Cardiovascular death***				
**MCA stenosis**	35	13.91(8.74–15.15)	3158.59	1.11
**No MCA stenosis**	120	14.61(13.07–15.66)	25192.88	0.48

Abbreviations: ACS, acute coronary syndrome; IQR, interquartile range; MCA, middle cerebral artery.

*Derived from median follow-up period (25^th^ to 75^th^ quartile).

† Derived from event rate per 100 person-years.

**Figure 1:**
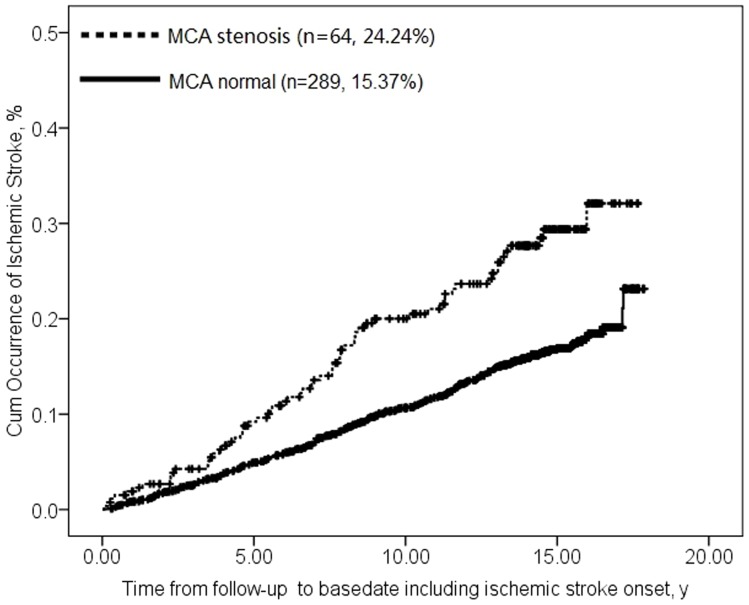
Survival plots of examining the association between asymptomatic MCA stenosis and ischemic stroke. Data for type 2 diabetic participants without MCA stenosis are shown with a solid line; data for type 2 diabetic participants with MCA stenosis are shown with a dashed line. (log rank χ^2^ = 21.27, P<0.001).

Stroke occurred in 64 individuals with MCA stenosis (24.2%, 64/264) and in 289 individuals without stenosis (15.4%, 289/1880) (P<0.001). Cumulative incidence of stroke at 5, 10, 15 and 18 years was 9%, 20%, 30% and 31% among participants with MCA stenosis; incidence seemed to plateau near 32%. The corresponding incidences among individuals without MCA stenosis were much lower: 5%, 11%, 17% and 24% (log rank χ^2^ = 21.27, P<0.001; Figure 1).

**Figure 2:**
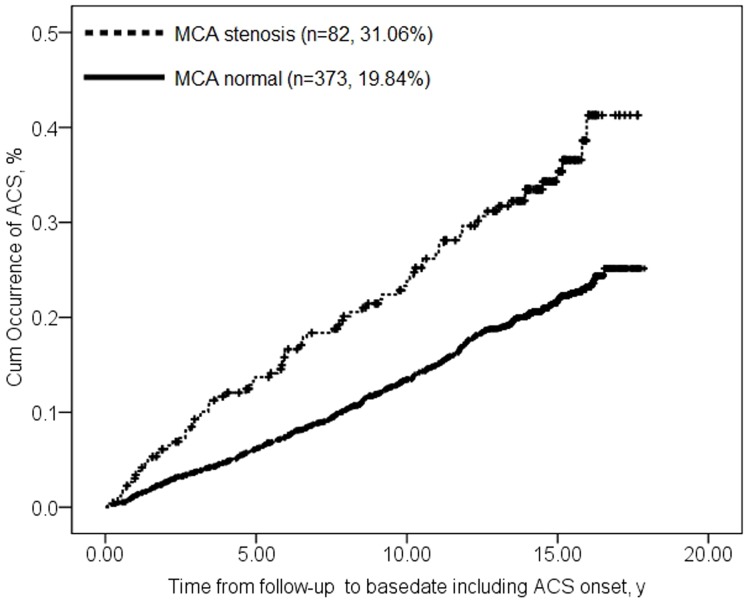
Survival plots of examining the association between asymptomatic MCA stenosis and acute coronary syndrome. Data for type 2 diabetic participants without MCA stenosis are shown with a solid line; data for type 2 diabetic participants with MCA stenosis are shown with a dashed line. (log rank χ^2^ = 26.87, P<0.001).

ACS occurred in 82 individuals with MCA stenosis (31.1%, 82/264) and in 373 without it (19.8%, 373/1880; P<0.001). Cumulative incidence of ACS at 5, 10, 15 and 18 years among those with MCA stenosis was, respectively, 14%, 24%, 35% and 40%; incidence seemed to plateau at 40%. The corresponding values for those without MCA stenosis were significantly lower: 6%, 13%, 22% and 25% (log rank χ^2^ = 26.87, P<0.001; Figure 2).

**Figure 3:**
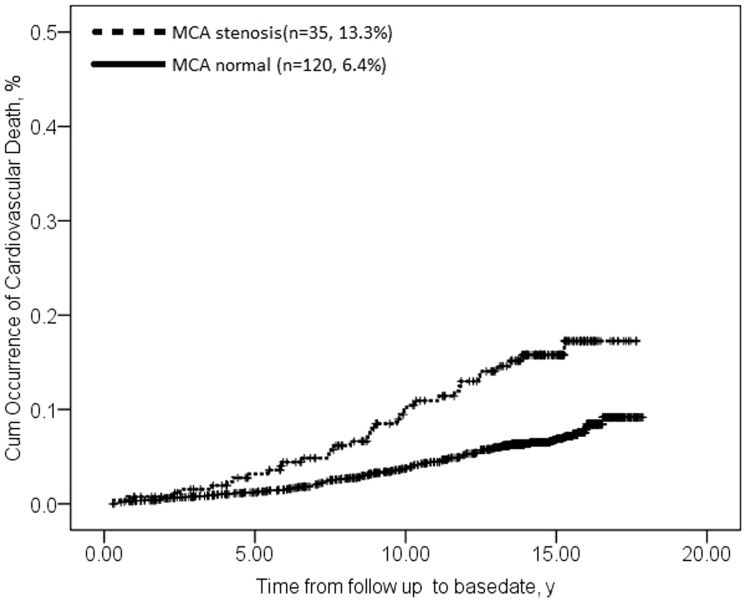
Survival plots of examining the association between asymptomatic MCA stenosis and cardiovascular death. Data for type 2 diabetic participants without MCA stenosis are shown with a solid line; data for type 2 diabetic participants with MCA stenosis are shown with a dashed line. (log rank χ^2^ = 22.516, P<0.001).

Cardiovascular death occurred in 35 cases with MCA stenosis (13.3%, 35/264) and in 120 cases without stenosis (6.4%, 120/1880) (P<0.001). Cumulative incidence of cardiovascular death at 5, 10, 15 and 18 years among individuals with MCA stenosis was, respectively, 3%, 10%, 17% and 19%; incidence seemed to plateau at 19%. The corresponding values for those without stenosis were significantly smaller: 1%, 4%, 7% and 9% (log rank χ^2^ = 22.516, P<0.001; Figure 3).

### MCA Stenosis and CVD Risk

Univariate analyses showed that individuals with asymptomatic MCA stenosis were at higher risk of stroke, ACS and cardiovascular death than were those without MCA stenosis (Table 3, model 1). Multivariable regression showed that asymptomatic MCA stenosis was associated with significantly higher risk of stroke and of ACS, while it was associated with moderately higher risk of cardiovascular death (HR 1.50, 95%CI 1.00–2.25; P = 0.05) (Table 3, model 2). These results were obtained after adjusting for age, sex, smoking, hypertension, total cholesterol, LDL, HDL, PAD, albuminuria, IHD history, diabetes duration, retinopathy, and HbA1C. Given recall bias and possible subjectivity in determining diabetes duration, we repeated the multivariable analysis after adjusting for the abovementioned factors except for diabetes duration (Table 3, model 3). The association between asymptomatic MCA stenosis and elevated risk of stroke and ACS remained significant. In addition, MCA stenosis was now found to be associated with significantly higher risk of cardiovascular death (HR 1.56, 95%CI 1.04–2.33; P = 0.03).

**Table 3:** Association between MCA stenosis and risk of cardiovascular events in Chinese with type 2 diabetes.

Event	Model 1*		Model 2†		Model 3‡	
	HR(95% CI)	P value	HR(95% CI)	P value	HR(95% CI)	P value
**Stroke**	1.87(1.43–2.46)	<0.001	1.40 (1.05–1.86)	0.02	1.40 (1.05–1.86)	0.02
**ACS**	1.86(1.47–2.37)	<0.001	1.34(1.04–1.74)	0.03	1.35(1.04–1.75)	0.02
**Cardiovascular death**	2.42(1.66–3.52)	<0.001	1.50(1.00–2.25)	0.05	1.56(1.04–2.33)	0.03

Abbreviations: HR, hazard ratio; MCA, middle cerebral artery.

HRs were calculated for participants with MCA stenosis with respect to participants without stenosis.

*Modle 1: Univariable analysis of MCA stenosis using a Cox proportional hazards model.

† Model 2: Cox proportional hazards model adjusted for age, sex, smoking, hypertension, total cholesterol, LDL, HDL, peripheral artery disease, albuminuria, IHD history, diabetes duration, retinopathy, and HbA1C.

‡ Model 3: Cox proportional hazards model adjusted for all the above mentioned factors except diabetes duration.

## Discussion

In this prospective, long-term cohort study, we evaluated the association between asymptomatic MCA stenosis and future cardiovascular events among Chinese with type 2 diabetes. Our findings indicate that asymptomatic MCA stenosis significantly increases risk of stroke, ACS and cardiovascular death. This suggests that detecting MCA stenosis by TCD can help identify individuals with diabetes at higher risk of these outcomes.

Among Chinese, middle cerebral artery (MCA) stenosis is the most commonly identified intracranial atherosclerotic vascular lesion [4]. Intracranial large-artery atherosclerosis (ILAA) is a major cause of ischemic stroke worldwide [15]; it involves progressive narrowing of intracranial arteries, leading to arterial stenosis. Carotid artery atherosclerosis contributes to stroke when stenosis exceeds 50% [16]. A recent community-based study in China showed large-artery intracranial occlusive disease to be more prevalent than carotid artery stenosis [17]. This suggests that atherosclerosis may begin in intracranial arteries and give rise to carotid artery stenosis only after progressing and becoming systemic. The same study also showed no significant association between the occurrence of intra- and extra-cranial artherosclerosis [17]. Therefore we focused on MCA stenosis in our participants and did not collect data on extra-cranial atherosclerosis.

Intracranial large-artery occlusive disease has already been linked to cardiovascular disease and death. Diabetes conferred a higher risk for Intracranial atherosclerotic disease than for extracranial atherosclerotic disease [18]. In a study of Chinese patients who experienced acute cerebral ischemia, the presence of occlusive vessels was the strongest predictor of vascular events or death within 6 months [4]. A study of Chinese with type 2 diabetes showed asymptomatic MCA stenosis to be an independent predictor of vascular mortality, including macro- and microvascular events [9]. That study did not, however, examine the relationship between MCA stenosis and cardiovascular death. The present work confirms and extends those studies by showing MCA stenosis to be an independent predictor of cardiovascular death, even after adjusting for a wide range of conventional vascular disease risk factors.

Studies in Hong Kong Chinese have suggested that atherosclerotic vascular disease may predict ischemic stroke in individuals with type 2 diabetes [19], and studies in Germany have reported that a large proportion of the general patient population with symptomatic MCA stenosis suffer ischemic stroke recurrence [6]. Previous work in Spain reported that stenosis progression independently predicts stroke recurrence in the general patient population with symptomatic MCA stenosis [20]. Studies in Chinese with asymptomatic MCA stenosis indicate that they are at 0.5% risk of ischemic stroke during the first year after stroke onset and at 1.6% risk during the second year [7]. The present study confirms and extends this previous work by showing that asymptomatic MCA stenosis is strongly associated with elevated risk of ischemic stroke in individuals with type 2 diabetes.

Our finding that Chinese with type 2 diabetes and asymptomatic MCA stenosis are at greater risk of ischemic stroke, ACS, and cardiovascular death runs against the conventional wisdom that asymptomatic MCA stenosis is benign in Caucasian and Asian patients [6], [7]. Previous studies in Germany and China reported that plaques in individuals with asymptomatic MCA stenosis were stable, and that stenosis in those individuals followed a benign course, leading to low annual incidence of TIA and stroke [6], [7]. In contrast, we found that macrovascular complications (e.g. ischemic stroke, ACS, and cardiovascular death) and microvascular complications had occurred in more of our subjects with asymptomatic MCA stenosis than in those without stenosis. In addition, the average yearly incidence of stroke in our cohort of Chinese with diabetes and asymptomatic MCA stenosis was higher than that reported for a general population of Chinese patients with asymptomatic MCA stenosis [7]. These findings support the notion that asymptomatic MCA stenosis can independently predict the occurrence of cardiovascular events in individuals with type 2 diabetes.

Inflammatory processes may underlie the observed association between asymptomatic MCA stenosis and cardiovascular events. Chronic inflammation plays a significant, independent role in the onset and development of both atherosclerosis and type 2 diabetes [21]. Inflammation is known to play a crucial role in all stages of atherogenesis, from early lesion formation to plaque progression and destabilization. Indeed, atherosclerosis is considered a systemic inflammatory disease. C-reactive protein (CRP), a sensitive indicator of systemic inflammation, has been shown to be a powerful predictor of future coronary and cerebral ischemic events, whether first-ever [22], [23] or recurrent [24], [25]. CRP level may also predict the progression of atherosclerosis [26]. In fact, elevated CRP levels within coronary plaques may contribute to the development and progression of atherosclerosis [27]: instability of coronary atherosclerotic plaques is the major cause of ACS [28].

If inflammation helps to explain the association between asymptomatic MCA stenosis and cardiovascular events, then diabetes is likely to strengthen this link. Individuals with type 2 diabetes exhibit a chronic inflammatory response. These individuals tend to have higher CRP concentrations than those without diabetes, and elevated CRP in diabetes is associated with increased risk of non-fatal cardiovascular events and cardiovascular death [29], [30]. Diabetes has been shown not only to enhance atherogenesis but also to promote vascular inflammation, plaque instability and clinical sequelae [31]. Therefore we speculate that diabetes may increase the occurrence of vulnerable plaques and promote the progression of asymptomatic MCA stenosis and coronary atherosclerosis, thereby increasing the incidence of ischemic stroke, ACS and cardiovascular death above the rates in the absence of diabetes.

Asymptomatic MCA stenosis may not increase the risk of different cardiovascular outcomes to the same extent. Among individuals with such stenosis in our cohort, cumulative and average yearly incidence were higher for ACS than for stroke. We suggest that atherosclerotic vascular lesions may be more tightly associated with the development of ACS than of stroke. This implies that individuals with type 2 diabetes and MCA stenosis are more vulnerable to ACS than to stroke, which is consistent with prior reports [32], [33].

The principal strengths of this study are that it reports a strong association between asymptomatic MCA stenosis and cardiovascular events in diabetic individuals based on a large sample size, long-term follow-up and complementary types of survival analysis. This is, to our knowledge, the first prospective cohort study on the impact of asymptomatic MCA stenosis on cardiovascular events.

At the same time, our study has several important limitations. First, it is a single-center, hospital-based study, so the generalizability of these findings needs to be validated. Second, although this study revealed important associations between asymptomatic MCA stenosis and CVD, it cannot address causation. A large body of evidence suggests that MCA stenosis is only one element in a multifactorial pathophysiological process that leads to CVD. Third, although we adjusted for multiple potential confounders in our analyses, we cannot exclude residual confounding by imperfectly measured or unmeasured factors, such as waist hip ratio or drug use during follow-up. Last but not least, when evaluating the effects of different treatment on CVD outcomes, we used the treatments at the baseline and assumed that the patients kept using the drugs during the whole period since it is difficult to follow up the changes of medication in such a big group of patients during a median of 15 years. Considering that there could be some changes of the treatments during the study period, our present findings may not accurately evaluate the effects of specific drug.

In conclusion, this study suggests that asymptomatic MCA stenosis enhances risk for future cerebrovascular and cardiovascular events in Chinese with type 2 diabetes. It also suggests that asymptomatic MCA stenosis plays a more important role in ACS than in stroke. One clinical implication of these findings is that stratifying diabetic patients based on the presence of asymptomatic MCA stenosis may help guide decisions to initiate primary prevention measures such as antiplatelet or statin therapy. This possibility may merit testing in clinical trials.
